# A Machine Learning Application to Classify Patients at Differing Levels of Risk of Opioid Use Disorder: Clinician-Based Validation Study

**DOI:** 10.2196/53625

**Published:** 2024-06-04

**Authors:** Tewodros Eguale, François Bastardot, Wenyu Song, Daniel Motta-Calderon, Yasmin Elsobky, Angela Rui, Marlika Marceau, Clark Davis, Sandya Ganesan, Ava Alsubai, Michele Matthews, Lynn A Volk, David W Bates, Ronen Rozenblum

**Affiliations:** 1 School of Pharmacy, Massachusetts College of Pharmacy and Health Sciences, Boston, MA, United States; 2 Division of General Internal Medicine, Brigham and Women's Hospital, Boston, MA, United States; 3 Innovation and Clinical Research Directorate, Lausanne University Hospital (CHUV), Lausanne, Switzerland; 4 Medical Directorate, Lausanne University Hospital (CHUV), Lausanne, Switzerland; 5 Harvard Medical School, Boston, MA, United States; 6 Vanderbilt University, Nashville, TN, United States; 7 Alexandria University, Alexandria, Egypt; 8 Clinical Quality and IS Analysis, Mass General Brigham, Somerville, MA, United States; 9 Department of Pharmacy, Brigham and Women's Hospital, Boston, MA, United States; 10 Harvard TH Chan School of Public Health, Boston, MA, United States

**Keywords:** opioid-related disorders, opioid use disorder, machine learning, artificial intelligence, electronic health record, clinical decision support, model validation, patient medication safety, medication safety, clinical decision, decision making, decision support, patient safety, opioid use, drug use, opioid safety, medication, OUD, EHR, AI

## Abstract

**Background:**

Despite restrictive opioid management guidelines, opioid use disorder (OUD) remains a major public health concern. Machine learning (ML) offers a promising avenue for identifying and alerting clinicians about OUD, thus supporting better clinical decision-making regarding treatment.

**Objective:**

This study aimed to assess the clinical validity of an ML application designed to identify and alert clinicians of different levels of OUD risk by comparing it to a structured review of medical records by clinicians.

**Methods:**

The ML application generated OUD risk alerts on outpatient data for 649,504 patients from 2 medical centers between 2010 and 2013. A random sample of 60 patients was selected from 3 OUD risk level categories (n=180). An OUD risk classification scheme and standardized data extraction tool were developed to evaluate the validity of the alerts. Clinicians independently conducted a systematic and structured review of medical records and reached a consensus on a patient’s OUD risk level, which was then compared to the ML application’s risk assignments.

**Results:**

A total of 78,587 patients without cancer with at least 1 opioid prescription were identified as follows: not high risk (n=50,405, 64.1%), high risk (n=16,636, 21.2%), and suspected OUD or OUD (n=11,546, 14.7%). The sample of 180 patients was representative of the total population in terms of age, sex, and race. The interrater reliability between the ML application and clinicians had a weighted kappa coefficient of 0.62 (95% CI 0.53-0.71), indicating good agreement. Combining the high risk and suspected OUD or OUD categories and using the review of medical records as a gold standard, the ML application had a corrected sensitivity of 56.6% (95% CI 48.7%-64.5%) and a corrected specificity of 94.2% (95% CI 90.3%-98.1%). The positive and negative predictive values were 93.3% (95% CI 88.2%-96.3%) and 60.0% (95% CI 50.4%-68.9%), respectively. Key themes for disagreements between the ML application and clinician reviews were identified.

**Conclusions:**

A systematic comparison was conducted between an ML application and clinicians for identifying OUD risk. The ML application generated clinically valid and useful alerts about patients’ different OUD risk levels. ML applications hold promise for identifying patients at differing levels of OUD risk and will likely complement traditional rule-based approaches to generating alerts about opioid safety issues.

## Introduction

In the past few decades, the “opioid epidemic” has become a public health crisis. According to a 2020 US survey, 2.7 million people aged 12 years or older had an opioid use disorder (OUD), and only 1 in 9 (11.2%) received medication-assisted therapy [1]. OUD is a frequently underdiagnosed condition, and it is estimated that for every patient with an OUD diagnosis, there are at least 2 who remain undiagnosed [2]. In 2021, nearly 92,000 drug overdose deaths were reported in the United States [3]. Furthermore, 54% and 46% of the US $1.02 trillion aggregate annual societal costs in 2020 in the United States were attributed to overdose deaths and OUD, respectively [4].

There is an immediate urgency to identify patients at high risk of OUD and those with OUD. Clinicians have reported major barriers to adequately assessing patients’ risk, including time pressure, incomplete or restricted medical records, and a lack of robust clinical decision support systems (CDSSs) [5,6]. The current rule-based approaches, such as Medicare Part D’s Overutilization Monitoring System or statewide Prescription Drug Monitoring Programs, fail to incorporate clinical data and are often underused [7]. Moreover, unless CDSSs use individual patient-specific clinical data in generating alerts, many false positive alerts may be presented to clinicians contributing to alert fatigue [8].

Artificial intelligence and machine learning (ML) algorithms have recently demonstrated their usefulness in CDSSs; however, compared with conventional statistical methods, their black-box nature and a lack of studies assessing the clinical validity of these interventions have created uneasiness in the medical community [9-12]. MedAware is a commercial software application that uses various statistical and ML methods to identify and prevent medication safety issues, including the risk of OUD [13]. It uses an iterative development process and has conducted pilot testing to optimize its OUD risk prediction algorithm to increase its accuracy in patient risk identification.

The goals of this study were to assess the clinical validity of the ML application by (1) determining the agreement between the ML algorithm’s output and the outcomes of structured clinicians’ review of medical records in classifying patients into distinct categories of OUD risk, including not high risk, high risk, or suspected OUD or OUD; (2) determining the potential utility of using the ML application as an alerting tool by evaluating its test characteristics against the gold standard; and (3) identifying major factors contributing to discrepancies between the ML application and clinician risk assignments to provide a knowledge base for future system improvement.

## Methods

### Ethical Considerations

This study was approved by the Mass General Brigham Institutional Review Boards (#2014P002167) that granted a patient waiver of consent for this study. Patients did not receive any compensation.

### Evaluation of the ML Application

MedAware (Ra’anana, Israel) has developed an ML software application to identify prescription errors and adverse drug events [13]. This application identifies medication issues based on ML methods including random forest algorithms—a widely used ML method in medical applications [14]. Multiple studies using ML models for disease prediction have achieved robust performance [15,16].

Based on clinical data in the electronic health record (EHR), the ML application’s algorithms generate patient-specific alerts on medication orders that deviate from predominant prescribing patterns in similar patient situations. Previously, it was found that the ML application generates medication error alerts that might otherwise be missed with existing applications with a high degree of alert usefulness, and it has the potential to reduce costs [17,18].

The ML application has been enhanced to generate alerts in real time to identify patients at risk of OUD and overdose based on clinical, psychosocial, and medication data. The input features used in the model were age, gender, opioid and nonopioid medication history (for each prescription: drug name, route of administration, duration, and dosage), and diagnosis history found in *ICD-9* (*International Classification of Diseases*) diagnoses codes and problem lists. The application can also produce aggregate alert data about the risk of OUD or overdose, which may be used for population health management.

The model outcome was defined by MedAware by combining OUD diagnosis codes, medication use, and experts’ annotation. The test cohort was independent from the training set to avoid overfitting. Random data splitting was conducted to separate training (50%) and test (50%) sets. MedAware used a scikit-learn (1.2.0) implementation of the random forest algorithm. It was used in a cross-fold manner and some of its hyperparameters (mainly: n_estimators, max_depth, class_weight) were tuned for optimization while leaving others at their default values. Additional details of the ML algorithm were not available to the research team because of intellectual property protections and were not the focus of this study; our study aimed to clinically validate OUD alerts generated by the algorithm against clinician judgement.

### Study Setting and Patient Population

The patient population of this study comprised patients who had at least 1 outpatient encounter between January 1, 2012, and December 31, 2013, and were prescribed at least 1 opioid medication between January 1, 2010, and December 31, 2013, in an outpatient setting at 2 large academic medical centers in the United States. Patients diagnosed with cancer and those with incomplete data were excluded. Once a patient had a documented OUD diagnosis or started receiving opioid rehabilitation drugs (eg, suboxone, naltrexone, methadone, and bezitramide), any subsequent patient data were excluded from the analysis as the patient’s status was known.

The evaluated application classified patients into 3 levels of OUD risk: not high risk, high risk, and suspected OUD or OUD. Alerts to clinicians are generated for only the high risk and suspected OUD or OUD categories. The risk alerts are generated when a clinician initiates an opioid medication prescription. A short textual description is created by the application for each alert generated to explain why the alert fired, for example, *the patient has a long opioid sequence, concurrent benzodiazepines use*. This explanation enables clinicians to understand the general reasoning underlying the alert. To improve study efficiency, the validation study comprised a random sample of 60 patients from each risk category for a total of 180 cases for which a retrospective review was performed by clinicians [19].

### Data Collection and Transfer

Clinical and encounter data on the patient population from 2010 to 2013 were extracted and sent to MedAware, including demographics, diagnoses, problem lists, outpatient and inpatient encounters, encounter clinicians, clinician specialties, procedures, medications, allergies, vital signs, and selected blood test outcomes. Patient and clinician names and medical record numbers were removed from the data set, and a random study ID was assigned to each patient and clinician before the limited data set was sent through a secure transfer application (password-protected and encrypted) for analysis.

### Development of a Risk Classification Scheme and Pilot Testing

Evaluation criteria for risk assignment by clinicians using the clinical data were developed with an extensive review of established guidelines, such as those of the Centers for Disease Control and Prevention and *DSM-5* (*Diagnostic and Statistical Manual of Mental Disorders, Fifth Edition*), and risk factors for OUD through an iterative process in consultation with experts in the field of pain and opioid management [20-24]. The research clinicians and team reviewed the Centers for Disease Control and Prevention’s and *DSM-5* guidelines and created draft criteria based on these guidelines to reflect 3 levels of risk, and then these criteria were reviewed by 2 pain management experts (a physician and a pharmacist). After modifications, this risk classification scheme was piloted to evaluate its effectiveness and compatibility with the ML application. We conducted the pilot review of medical records with 25 randomly selected medical records. One research assistant (CD) extracted data from the medical records using a standardized data collection tool as described below and 2 physician reviewers (FB and TE) individually reviewed the data. The reviewers reached a consensus on their risk determinations, and revisions were made to criteria, as needed, to standardize assessments and support a more transparent, generalizable validation process. MedAware sent a list of those patients for whom a risk assessment was conducted to be used for selecting the random sample for review of medical records.

### Structured Clinicians’ Review of Medical Records Using a Standardized Data Collection Tool

**Figure 1.**
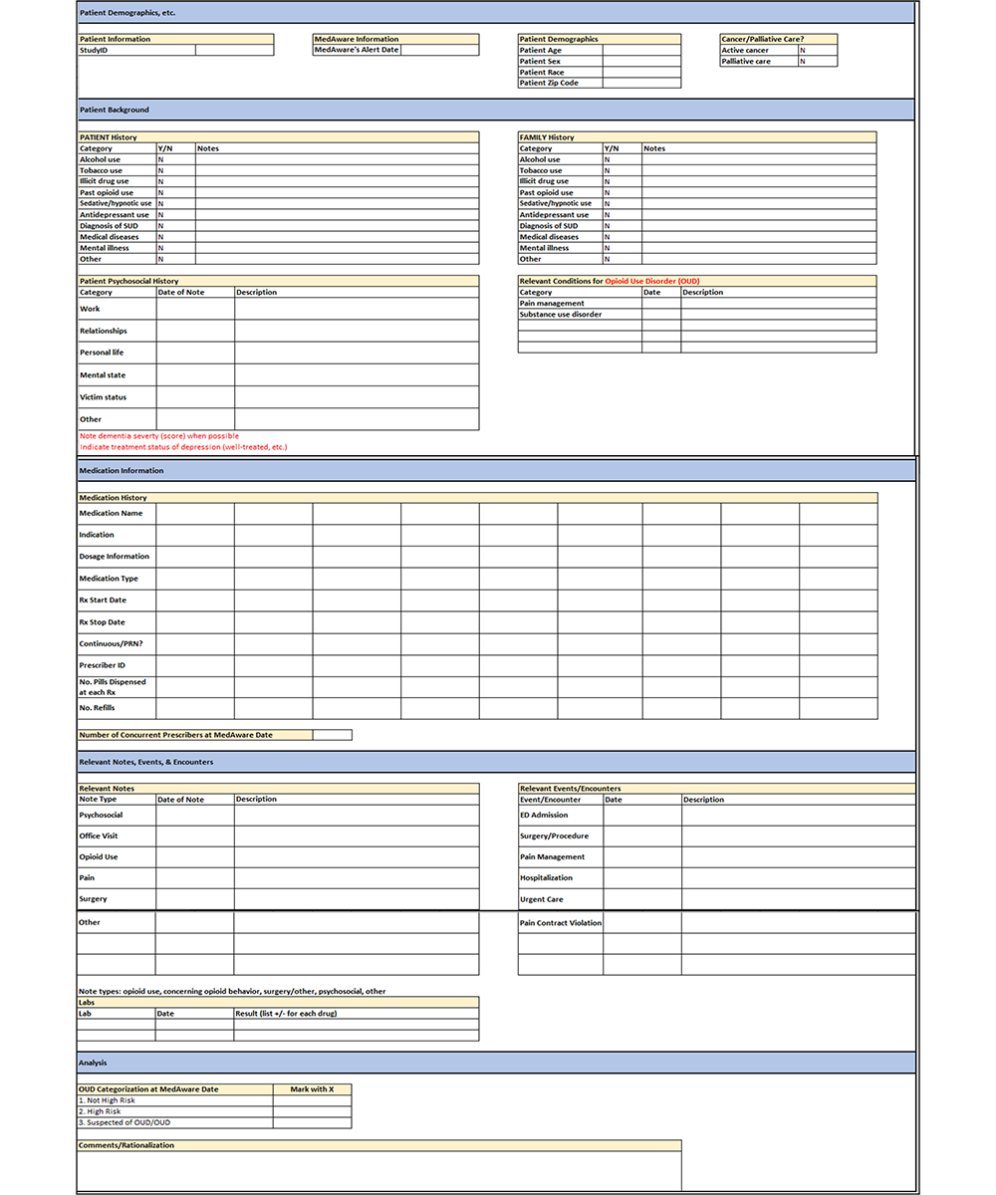
Tool used to extract data from patients’ medical records. Template used to organize patient information extracted during the review of electronic health records (EHRs). A patient’s demographics and relevant past medical, psychosocial, family, and medication histories were captured. Provider notes and encounters relevant to opioid use and pain management were described and recorded by date. Any patient’s laboratory findings relevant to opioid use or other medications of interest were also recorded. Clinician reviewers recorded their risk categorization and rationalization after reviewing the information captured on the data extraction tool and reviewing the EHR, as needed. OUD: opioid use disorder; PRN: pro re nata; SUD: substance use disorder.

**Figure 2.**
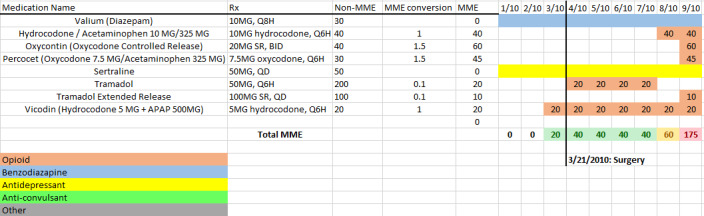
Example medication timeline from the tool used to extract data from the review of medical records. This timeline was created after medication history was recorded and morphine milligram equivalent (MME) conversion for opioid medications was included. Relevant encounters were recorded by date to provide context for the medication timeline (eg, surgery). Medications of interest included opioids, benzodiazepines, antidepressants, anticonvulsants, and other nonopioid medications contributing to risk. BID: twice a day; Q6H: every 6 hours; Q8H: every 8 hours; QD: every day, daily; SR: sustained release.

In total, 180 patients with a history of opioid use were randomly selected from those patients classified by the ML application into 3 risk categories (60 in each group), and structured reviews of medical records were conducted to evaluate patients’ OUD risk. Clinicians were blinded to the patients’ risk assignment by the application. A data abstraction tool was developed to organize relevant patients’ clinical data from an EHR and facilitate the process for the review of medical records (Figure 1). This tool contains important demographic, patient, and family medical history including psychiatric and psychosocial information, patient complaints as documented in relevant clinical notes, relevant laboratory findings and drug history with graphical representation of opioid drug start and stop dates (ie, medication timeline; Figure 2), clinical events relevant to pain management such as surgeries or dates of major accidents, admission and emergency room visits, and curated clinical notes related to relevant clinical events. Collected data included both structured and free-text data that were extracted by research staff and organized into the abstraction tool. Data collection was focused on relevant information during the 2010-2013 time period; however, as the complete medical record was available for review, relevant information available prior to 2010 may have been considered. After training, 5 research assistants (CD, AA, SG, AR, and MM) individually extracted clinical data. Information from medical records was reviewed by extractors and clinicians up to the ML application’s first alert date (index date). For patients determined to be not high risk by the ML application, a random date was assigned up to which medical record data were extracted and reviewed.

Four clinician reviewers (FB, TE, DM, and YE) individually examined data extracted by the research assistants and reviewed the EHRs directly, as needed, to holistically understand the clinical context of opiate prescription for the patients. The reviewers comprised 2 general internal medicine physicians, a hospitalist with extensive daily opioid prescribing experience, one with a PhD focusing on pharmacoepidemiology and drug safety, a recent medical student, and a pharmacist. All medical records were reviewed by 2 independent clinicians. After the primary review of medical records, a second reviewer blinded to the risk assignment of the first reviewer determined the risk level for OUD. The 2 clinicians discussed the case to reach consensus when their risk assignments differed. This consensus determination was then compared to the ML application’s alert. Statistical analyses were conducted to evaluate the level of agreement between the clinician reviewers and the ML application’s risk classifications.

### Evaluation of Reasons for Disagreement Between Risk Assignments

To evaluate and identify the main reasons for disagreement between the clinician reviewers and the ML application’s risk classifications, a qualitative analysis was also conducted. For cases where there was disagreement, additional information contributing to the system risk assessment was requested from MedAware. Using a thematic analysis approach, 3 members of the research team (AR, LAV, and MM) independently conducted a qualitative analysis of the alert information. They reviewed the ML application’s reasoning for assigning a particular risk category, information from the data extraction sheet, and information from the clinician reviewer’s final risk assignment consensus. Then, this information was systematically coded to identify, categorize, and sort key concepts for the disagreements. Codes were then grouped into emergent themes and relationships after iterative review and discussion. In cases where there was disagreement, all 3 researchers reviewed and discussed the case together to reach consensus.

### Statistical Analysis

We used descriptive statistics to summarize demographic characteristics of the study population, patients in each of the 3 risk categories identified by the ML application, and the 180 patients sampled for the validation study. We assessed the validity of the application by comparing them to the structured clinicians’ review of medical records. The agreements between the 2 methods were evaluated with the following parameters:

1. Overall percent agreements were calculated, including percent agreements for the 3 risk categories. Disagreements were reported for the overall validated sample and the 3 opioid risk categories.
2. Weighted kappa and 95% CIs were reported because of the ordered nature of the risk categories to measure the agreement between the 2 methods.
3. Naïve sensitivity and naïve specificity were calculated along with positive and negative predictive values for the ML application using the structured clinicians’ review of medical records as a gold standard and combining the 2 opioid risk categories, namely high risk and suspected OUD or OUD.
4. Corrected sensitivity and corrected specificity were calculated to account for verification bias, that is, overestimation of sensitivity and underestimation of specificity [19,25,26]. Verification bias occurs when disease status (eg, the presence or absence of OUD) is not ascertained in all participants by the gold-standard method (review of medical records) and proportionately more high risk and suspected OUD or OUD patients identified by the test methodology (eg, the ML algorithm) were selected for verification. This verification-biased sampling increases sensitivity and decreases specificity, and these parameters are mathematically corrected to adjust for the biased sampling method.
5. Descriptive statistics were calculated for evaluating risk assignments to determine the most frequently occurring themes for disagreement between the 2 methods.

## Results

### Patient Risk Categories and Demographics

**Figure 3.**
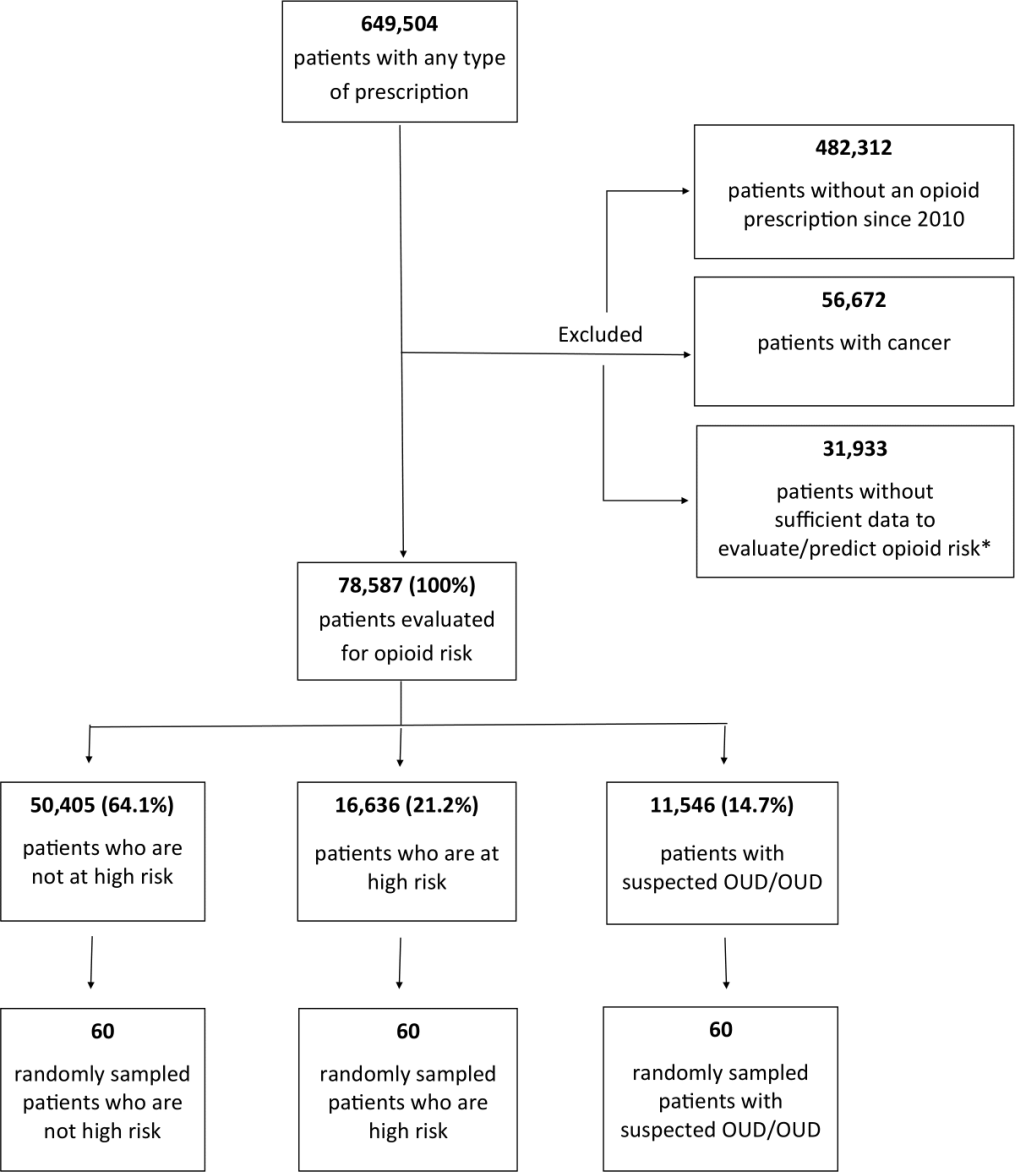
Patient flow diagram with the final verification sample. Patients were excluded from the overall population if they did not have any opioid prescriptions since 2010, were diagnosed with cancer, or had insufficient data to predict opioid risk. The remaining patients were evaluated for opioid risk and stratified by risk classification category. A total of 60 patients were randomly sampled from each risk classification category to be used for the review of medical records and clinician evaluation.

Of the 649,504 eligible patients with at least 1 prescription in the source data, 78,587 (12.1%) were classified by the ML application into the 3 risk categories after excluding patients with no opioid prescription, patients without sufficient data to evaluate opioid risk, or patients with a diagnosis of cancer (Figure 3). Patients were excluded due to insufficient data if they did not have 1 day before and 1 year of data after their first opioid prescription, or if they were identified as having OUD (based on a diagnosis or rehabilitation drug) and did not have a first opioid prescription before identification of OUD. Patients with opioids prescribed within 2 years of a cancer diagnosis based on *ICD-9* (*International Classification of Diseases, Ninth Revision*) codes were excluded. Accordingly, 50,405 (64.1%) patients were classified by the ML application as being in the not high risk category, 16,636 (21.2%) as being in the high risk category, and 11,546 (14.7%) as being in the suspected OUD or OUD category. We excluded patients who do not have 1 day before and 1 year of data after the first opioid Rx or, if identified as having OUD (based on diagnosis or rehabilitation drug) and do not have a first opioid Rx before identification.

Table 1 details the distribution of eligible patients by demographic characteristics across the different ML application risk assignment categories and sampled patients. Female sex and age 30-64 years were overrepresented in the groups with opioid prescriptions and validation samples for medical records review compared to the eligible patient pool. The sample randomly selected for validation with the structured review of medical records was representative of the patients on opioid treatment with regard to age, sex, and race.

**Table 1.** Demographic characteristics of the study populations, including the overall patient population, patients who met the criteria for opioid risk evaluation, patients stratified by the machine learning (ML) application’s risk categories “not high risk,” “high risk,” or “suspected OUD or OUD,” and for the validation sample for total review of medical records.

Patient characteristics		Patients with at least 1 prescription (n=649,504), n (%)	Patients meeting criteria for opioid risk evaluation (n=78,587), n (%)	Not high risk (n=50,405), n (%)	High risk (n=16,636), n (%)	Suspected OUD or OUD (n=11,546), n (%)	Sampled patients (n=180), n (%)
**Sex**							
	Female	385,959 (59.4)	50,064 (63.7)	31,184 (61.9)	11,860 (71.3)	7020 (60.8)	116 (64.4)
	Male	263,535 (40.6)	28,521 (36.3)	19,221 (38.1)	4775 (28.7)	4525 (39.2)	64 (35.6)
	Unknown	10 (0.0)	2 (0.0)	0 (0.0)	1 (0.0)	1 (0.0)	0 (0.0)
**Age (years)**							
	0‐17	76,024 (11.7)	737 (0.9)	635 (1.3)	77 (0.5)	25 (0.2)	4 (2.2)
	18‐29	83,216 (12.8)	8467 (10.8)	5953 (11.8)	1645 (9.9)	869 (7.5)	22 (12.2)
	30‐49	180,603 (27.8)	27,666 (35.2)	17,777 (35.3)	5834 (35.1)	4055 (35.1)	64 (35.6)
	50‐64	164,188 (25.3)	24,329 (31.0)	14,442 (28.7)	5564 (33.4)	4323 (37.4)	51 (28.3)
	≥65	145,473 (22.4)	17,388 (22.1)	11,598 (23.0)	3516 (21.1)	2274 (19.7)	39 (21.7)
**Race** ^a^							
	American Indian or Native Alaskan	719 (0.1)	89 (0.1)	60 (0.1)	16 (0.1)	13 (0.1)	0 (0)
	Asian	28,328 (4.4)	2211 (2.8)	1763 (3.5)	297 (1.8)	151 (1.3)	3 (1.7)
	Black or African American	41,794 (6.4)	6189 (7.9)	4119 (8.2)	1245 (7.5)	825 (7.1)	11 (6.1)
	Native Hawaiian or Pacific Islander	286 (0.0)	26 (0.0)	20 (0.0)	5 (0.0)	1 (0.0)	0 (0)
	White	475,939 (73.3)	58,617 (74.6)	36,972 (73.3)	12,373 (74.4)	9272 (80.3)	135 (75.0)
	Other (unknown, declined, or bi- or multiracial)	102,438 (15.8)	11,455 (14.6)	7471 (14.8)	2700 (16.2)	1284 (11.1)	31 (17.2)
**Ethnicity** ^a^							
	Hispanic or Latino	43,119 (6.6)	1874 (2.4)	1279 (2.5)	420 (2.5)	175 (1.5)	17 (9.4)
	Other^b^	606,385 (93.4)	76,713 (97.6)	49,126 (97.5)	16,216 (97.5)	11,371 (98.5)	163 (90.6)

a Race and ethnicity data are based on coded fields in the electronic health record.

b Other refers to non-Hispanic or non-Latino, declined to respond, and unknown.

### Percent Agreement and Kappa Statistics

Prior to conducting final consensus assessments, the independent clinician reviewers’ assessment of the levels of risk matched exactly for 70% (126/180) of the patients. When comparing assessments of the not high risk group and those of the high risk and suspected OUD or OUD groups, the clinician reviewer assessments matched 88% of the time.

The overall percent agreement between the ML application and clinician reviewers in stratifying patients into 3 risk categories was 70% (126/180 patients; Table 2). Of the 30% disagreements, 22.8% (n=41) and 7.2% (n=13) indicated underestimation and overestimation of risk by the ML application, respectively, compared to the clinicians’ structured review of medical records. Among different risk categories, percent agreement was the highest (90%) for the suspected OUD or OUD category than for the not high risk and high risk categories (60% each). Of the patients classified to the suspected OUD or OUD category by the ML application, 8.3% and 1.7% of them were classified to the high risk and not high risk categories, respectively, by the clinicians’ review of medical records. Of the patients classified to the not high risk category by the ML application, clinician reviews classified 40% of patients to the 2 higher risk categories: 30% of patients to the high risk category and 10% of patients to the suspected OUD or OUD category.

**Table 2.** Distribution in opioid risk assignment between the machine learning (ML) application and clinicians’ structured review of medical records of 180 randomly sampled patients (percent agreement 70%, 95% CI 63.3%-76.7%; weighted kappa coefficient 0.62, 95% CI 0.52-0.71).

ML system risk assignment	Clinician reviewer risk assignment, n			
Not high risk	High risk	Suspected OUD/OUD	Total
Not high risk	36	18	6	60
High risk	7	36	17	60
Suspected OUD^a^ or OUD	1	5	54	60

a OUD: opioid use disorder.

The interrater reliability, as expressed using the weighted kappa coefficient for the 2 methods, was 0.62 (95% CI 0.53-0.71), indicating good or substantial agreement [27].

### Corrected Sensitivity, Corrected Specificity, and Positive and Negative Predictive Values

Table 3 presents a revised version of Table 2, where the 2 higher-level opioid risk categories (high risk and suspected OUD or OUD) were combined to investigate the potential utility of the ML application in generating signals or alerts to prescribing clinicians, that is, how complete and accurate the ML application is in identifying patients who are at the risk of developing or who may already have OUD. The naïve sensitivity of the ML application was 82.4% (95% CI 75.9%-88.9%), and its naïve specificity was 81.8% (95% CI 70.2%-93.4%). After accounting for verification-biased sampling, the corrected sensitivity of the ML application was 56.6% (95% CI 48.7%-64.5%) and its corrected specificity was 94.2% (95% CI 90.3%-98.1%). The positive and negative predictive values of the ML application were 93.3% (95% CI 88.2%-96.3%) and 60.0% (95% CI 50.4%-68.9%), respectively.

**Table 3.** Distribution in opioid use disorder (OUD) risk assignment between the machine learning (ML) application and clinicians’ structured review of medical records when the 2 higher-risk categories were combined to investigate the utility of an OUD risk alert at the time of prescribing.

ML system risk assignment	Clinician reviewer risk assignment, n		
High risk and suspected OUD or OUD	Not high risk	Total
High risk and suspected OUD or OUD	112	8	120
Not high risk	24	36	60
Total	136	44	180

### Key Reasons for Disagreements in OUD Risk Categories Between the ML Application and Clinician Reviewers

Table 4 contains the 6 themes that emerged as reasons for disagreements between the ML application and the clinicians’ structured review of medical records after conducting a qualitative analysis. Disagreement between the 2 methods was noted for 54 patients, among whom the ML application underestimated the OUD risk in 41 patients and overestimated it in 13 patients. Two or more themes were identified as reasons for most of the disagreements (74.9%). Of the 6 themes, the theme “differences in risk assessment of medication information,” accounted for most of the disagreements (72%), followed by the theme “information in clinical notes not available to the ML application” (55.6%).

**Table 4.** Key reasons for disagreements in opioid use disorder (OUD) risk assignments between the machine learning (ML) application and clinician reviewers. The reasons for discrepancies were categorized into 6 major themes. More than 1 reason might be identified for a given patient. Results are displayed by whether the assigned risk category was underestimated or overestimated by the ML application in comparison with the clinician reviewers.

Themes of reasons for disagreements in OUD risk assignment	Description of the themes	Patients with at least 1 reason coded in a given theme category, n (%)		
		Cases underestimated by MedAware^a^ (n=41)	Cases overestimated by MedAware^b^ (n=13)	Total discrepant cases (n=54)
I. Differences in risk assessment of medication information	Medication information available to both the clinician reviewers and the MedAware system contributed to differing risk assessments (eg, medication duration, dose, indication, and gaps in medication timelines).	30 (73.2)	9 (69.2)	39 (72.2)
II. Information in clinical notes not available to MedAware system	Information in patients’ clinical notes was available to the clinician reviewers but not to the MedAware system (eg, psychosocial information, experience with opioids and other medications, patient participation in pain management and substance abuse services, and medication information not on the medication list).	27 (65.9)	3 (23.1)	30 (55.6)
III. Differences in risk assessment of psychosocial issues	Psychosocial or psychiatric information available to both the clinician reviewers and the MedAware system contributed to differing risk assessments (eg, patient history of substance abuse, family members with a history of psychosocial or psychiatric issues, and the presence of patients’ individual psychiatric conditions contributed to differing risk assessments).	17 (41.5)	2 (15.4)	19 (35.2)
IV. Differences in risk assessment of nonopioid medications	Information on nonopioid medications available to both research reviewers and the MedAware system, which reflects an increased complexity of the patient’s medical situation (eg, pain level) or a higher risk when combined with opioids, contributed to differences in risk assessments (eg, zolpidem and gabapentinoids).	10 (24.4)	2 (15.4)	12 (22.2)
V. Bugs identified in the MedAware system	Bugs in the MedAware system included inaccurate mapping of data elements (eg, dosage units and incorrect medication), missing medication in drug class, and incorrectly constructed alert messages.	5 (12.2)	5 (38.5)	10 (18.5)
VI. Presence of other clinical information not considered by the MedAware system or the clinician reviewers	Clinical information that may indicate the risk of OUD not considered by the clinician reviewers or the MedAware system, but not both, such as hepatitis C diagnosis, urine toxicity tests, and MedAware system access to *ICD-9*^c^ diagnostic information that clinician reviewers did not see.	6 (14.6)	0 (0.0)	6 (11.1)

a The ML application’s risk assignment was lower in severity compared to the clinician reviewers’ risk assignment.

b The ML application’s risk assignment was higher in severity compared to the clinician reviewers’ risk assignment.

c *ICD-9*: *International Classification of Diseases, Ninth Revision*.

## Discussion

### Principal Results

ML algorithms can leverage large-scale EHR and medical claims data and potentially identify patients at risk of OUD [28-32]. However, very few studies have assessed the clinical validity and potential utility of ML algorithms designed to differentiate among levels of patients’ OUD risk. In this study, we examined the agreement between an ML application and clinicians’ structured review of medical records in classifying patients on opioid drug treatment into 3 distinct categories of OUD risk (ie, not high risk, high risk, or suspected OUD or OUD). We also assessed the application’s utility in identifying clinically valid alerts and identified and quantified reasons that could lead to disagreements between clinicians’ judgment and outputs of ML applications. The ML application was validated in an outpatient database, and it appeared to have value.

There was substantial agreement between the application and the clinician reviewers’ structured review of medical records. The agreement between the 2 methods was the highest for the suspected OUD or OUD category. The ML application correctly identified this most vulnerable group of patients to increase clinician awareness and responsiveness to improve patient management, including modifications to their medication regimen or referral to a specialized treatment service to mitigate the complications of opioid use. Moreover, if the ML application is used to generate alerts on patients at high risk of OUD or those who already have OUD, it will identify approximately 60% of these patients with a 93.3% precision (positive predictive value). Thus, the results of this study show that this ML application was able to generate clinically valid and useful alerts to screen for patients at risk of OUD. It is important to recognize that alerting clinicians regarding patients at risk of OUD should be coupled with clinician education on appropriate treatment guidelines and practices to avoid undertreatment of pain and patient stigma [33,34].

### Comparison With Prior Work

Previous studies have shown that artificial intelligence tools using ML algorithms can improve treatment, enhance quality of care and patient safety, reduce burden on providers, and generally increase the efficiency with which resources are used, resulting in potential cost savings or health gains [7,32,35-38]. In addition, our findings align with those of previous studies that highlight the potential of ML applications to predict individual patients’ risk of specific medical conditions and associated complications to offer specialized care programs to high-risk patients [39,40]. Our study also confirms and extends the findings of a few studies that examined other ML applications and highlighted the potential to identify patients at risk for substance misuse and abuse, including OUD and opioid overdose [31,38,41]. Nevertheless, these comparable ML applications were plagued with very low positive predictive values due, in part, to low OUD prevalence as a result of suboptimal definitions of OUD by relying solely on *ICD* (*International Classification of Diseases*) codes [42]. A few previous studies identified additional limitations and challenges related to comparable ML applications. For example, Afshar et al [43] described the use of an algorithm to identify patients at risk for any substance misuse at the time of admission, based on clinical notes from the first 24 hours after hospital admission. In this study, we found that the positive predictive value of this tool was 61%-72%, which was lower than that of the ML application. The tool that Afshar et al [43] studied does not identify patients outside of the hospital setting and depends on physicians’ notes. As a result, this tool is not suited for more general screening using structured clinical EHR data and medical claims data. Another recent study by Lo-Ciganic et al [41] described an algorithm to predict the occurrence of overdose episodes, but does not identify patients who are most at risk of OUD in the future.

We believe that the substantial agreement, high specificity, and high positive predictive value of the ML application was achieved because we pilot-tested the ML models in comparison with clinician assessments and then used an iterative process with continuous calibration of model parameters to optimize the accurate identification of OUD risk categories. In addition, we used a composite definition of OUD not restricted to *ICD* codes resulting in a higher prevalence of OUD identified in the patient population. The ML application classified 1 in 7 and about one-fifth of the eligible population with prescribed opioids in the suspected OUD or OUD and high risk categories, respectively, compared to other studies that reported a prevalence of OUD in the range of 1%-5% [44,45]. Furthermore, the full accessibility of the EHR at the time of case evaluation, coupled with standardized data extraction and a medication timeline visualization tool, allowed seamless analysis of cases contributing to the high accuracy rates.

Our study also identified the main reasons for disagreements between the clinician reviewers and the ML application’s risk assignments. These reasons included information available in the clinical notes not being accessible to the ML application (eg, psychosocial issues and patients’ participation in substance abuse services), and different interpretation of available information such as differences in the impact of antidepressant treatments. Clinicians considered stable and sufficiently treated depression as not being a risk factor for OUD [46]. In analyzing the reasons for discrepancies, we observed factors related to model training processes, data quality, and outcome definitions. The knowledge gained through our analytic process could be useful to further optimize their ML algorithm development pipeline. As of today, it is critical to standardize the ML development process and make it more understandable to clinical end users. However, to our knowledge, few efforts have been made to systematically analyze each component of the model development process from the clinician’s point of view and further evaluate its impact on the model’s clinical implementation. We believe that our work can facilitate a better bridging of the gap between ML model builders and clinicians.

### Limitations

Our study has some limitations. We used retrospective data to evaluate an algorithm primarily designed to be used in real time. Although many of the findings from our retrospective analysis should be applicable to real-time alerting, it is difficult to predict whether some alerts would perform differently or how clinicians would respond to real-time alerts. Second, although our clinician reviewers were carefully trained and a coding manual was developed with clear operational definitions, each risk assessment required a degree of judgment on the part of the reviewers; human factors could impact the final risk assignment. Finally, our study was limited to outpatients at 2 large academic medical centers in the United States, which limits the generalizability of our results. Additional biases may have been introduced into the ML application in ways that the research team were not able to assess [7,47]. Although the total population of patients receiving outpatient care within an academic medical center was included, there may have been biases in patients who were able to access care, those receiving opioid prescriptions, and in the clinical documentation of concerns regarding opioid use and substance abuse. Validation across different sites and populations (eg, veterans’ facilities) may reveal site-specific differences and may require unique models or warrant the identification and capture of new descriptive features.

### Conclusions

We tested an ML application that assessed OUD risk in an extensive outpatient EHR database and found that it appeared to classify patients into differing levels of OUD risk, and that there was substantial agreement with clinicians’ review of medical records. We identified key themes for disagreements between the commercial application and clinician review, which can be used to further enhance ML applications. ML algorithms applied to available EHR clinical data hold promise for identifying patients at differing levels of OUD risk and supporting better clinical decision-making regarding treatment. Such tools will likely complement traditional, rule-based approaches to provide alerts about potential opioid prescribing safety issues.

## Abbreviations

CDSS: clinical decision support system
DSM-5: Diagnostic and Statistical Manual of Mental Disorders, Fifth Edition
EHR: electronic health record
*ICD*: *International Classification of Diseases*
*ICD-9*: *International Classification of Diseases, Ninth Revision*
ML: machine learning
OUD: opioid use disorder
